# Prediction of stress-related gastrointestinal bleeding in patients with aneurysmal subarachnoid hemorrhage using caudate Hounsfield unit value in ASPECT score

**DOI:** 10.3389/fneur.2023.1237310

**Published:** 2023-09-13

**Authors:** Ke Wang, Kexin Yuan, Runting Li, Fa Lin, Yu Chen, Jun Yang, Heze Han, Tu Li, Yitong Jia, Yunfan Zhou, Haibin Zhang, Ruinan Li, Zhipeng Li, Yahui Zhao, Qiang Hao, Xiaolin Chen, Yuanli Zhao

**Affiliations:** ^1^Department of Neurosurgery, Beijing Tiantan Hospital, Capital Medical University, Beijing, China; ^2^Stroke Center, Beijing Institute for Brain Disorders, Beijing, China; ^3^Beijing Key Laboratory of Translational Medicine for Cerebrovascular Disease, Beijing, China; ^4^China National Clinical Research Center for Neurological Diseases, Beijing, China

**Keywords:** aneurysmal subarachnoid hemorrhage, stress-related gastrointestinal bleeding, ASPECT score, Hounsfield units, caudate

## Abstract

**Background:**

Stress-related gastrointestinal bleeding (SRGB) is one of the major complications after aneurysmal subarachnoid hemorrhage (aSAH), and it can present challenges in patient care and treatment. The aim of this study was to explore the clinical significance of the caudate Hounsfield unit (HU) value in the Alberta Stroke Program Early CT (ASPECT) score for predicting SRGB in patients with aSAH.

**Methods:**

We retrospectively analyzed the data of 531 aSAH patients admitted to our institution between 2019 and 2022. Potential predictors of SRGB were identified using multivariate Cox regression analysis. We used a restricted cubic spline (RCS) to evaluate whether there is a nonlinear relationship between the right caudate HU value and SRGB. MaxStat analysis (titled as maximally selected rank statistics) was performed to identify the optimal cutoff point for the right caudate HU value. Another Kaplan–Meier method with the log-rank test was used to analyze the right caudate HU value in predicting the occurrence of SRGB.

**Results:**

The incidence rate of SRGB was 17.9%. In the multivariate Cox regression analysis, the right caudate HU value was an independent predictor of SRGB [Hazard ratio (HR) = 0.913; 95% confidence interval (CI): 0.847–0.983, and *p* = 0.016]. The RCS indicated that the incidence of developing SRGB reduces with increasing right caudate HU values (nonlinear *p* = 0.78). The optimal cut-off value of the right caudate HU was 25.1.

**Conclusion:**

Among aSAH patients, lower right caudate HU values indicated a higher risk of developing SRGB. Our findings provide further evidence for the relationship between the gastrointestinal system and the brain.

## 1. Introduction

Aneurysmal subarachnoid hemorrhage (aSAH) is typically characterized by extravasation of blood into the subarachnoid space and is induced by the spontaneous rupture of intracranial aneurysms. aSAH accounts for approximately 85% of atraumatic subarachnoid hemorrhages and is a crucial cause of morbidity and mortality worldwide (1). At the very minimum, a quarter (25%) of aSAH patients die, while around half (50%) of the survivors will experience prolonged psychological and neurological deficits (2).

In addition to causing severe impairment to the central nervous system, aSAH also has deleterious effects on various organs, including the gastrointestinal and cardiovascular systems. These effects can pose challenges to the provision of patient care and treatment (3). Stress-related mucosal disease accompanies a state of severe stress, such as critical illnesses and severe trauma. It results in injury to the superficial or deep local mucosal layers in the form of stress-related mucosal injury or stress ulcers, respectively. About 5–33% of stress-related gastrointestinal bleeding (SRGB) is clinically significant as it leads to hemodynamic instability, heightened ventilation requirements, elevated rates of hospitalization, and augmented mortality rates (4–6).

Numerous studies have demonstrated a prominent role of the dopaminergic system in specific gastrointestinal functions (7, 8). In addition, stress affects both central and peripheral dopaminergic activity, thereby further regulating the effects of stress-induced gastroduodenal mucosal injury. As one of the three principal dopaminergic pathways in the central nervous system, the nigrostriatal pathway primarily comprises cell bodies in the substantia nigra pars compacta (SNpc), which project to the caudate putamen and the central nucleus of the amygdala. Studies have reported that damage to the nigrostriatal pathway could promote the development of gastric erosions (9, 10).

Computed tomography (CT), as a reliable and safe indicator, is being increasingly applied to clinical research pertaining to both cerebral ischemic and hemorrhagic diseases. The Hounsfield unit (HU) value serves as an indicator of water uptake in infarcted tissue. Increased water absorption leads to a decrease in the HU value (11). Studies have established a correlation between the HU value and cerebral vasospasm or delayed cerebral ischemia in patients affected by aSAH (12). The Alberta Stroke Program Early Computer Tomography (ASPECT) score employs a scoring method that evaluates early ischemic changes on CT scans in the areas of the brain supplied by the middle cerebral artery (MCA). It also computes the mean HU value of the region containing the caudate, internal capsule, lentiform nucleus, and insula. Currently, the ASPECT score can be automatically generated by an artificial intelligence system known as Rapid, which acts as a bridge for assessing the relationship between the caudate and SRGB.

Elevated intracranial pressure post-aSAH can cause a decline in cerebral perfusion pressure. Thus, we aimed to determine whether a relationship between the caudate and SRGB exists using the HU value in the ASPECT score.

## 2. Materials and methods

### 2.1. Patient selection

We retrospectively analyzed the data of aSAH patients admitted to our institution between January 2019 and September 2022. Pertinent data related to these patients were extracted from the Long-Term Outcomes in Emergency Aneurysmal Subarachnoid Hemorrhage (LongTEAM) study, which has been officially registered at ClinicalTrials.gov (registration number: NCT 04785976). This study received ethical approval from the Institutional Review Board of Beijing Tiantan Hospital (KY 2021–008-01), and written informed consent was obtained from each patient at admission before gathering any clinical information. All included patients received medical attention and care based on the standards recommended by the American Heart Association/American Stroke Association (13).

The inclusion criteria were as follows (1): age ≥ 18 (2); emergency admission (due to aneurysm rupture <72 h after admission, and time from admission to treatment <72 h) (3); single aneurysm; and (4) non-contrast CT scanning was performed and the ASPECT score was obtained before treatment. The exclusion criteria were as follows (1): physical disability and functional deficits due to any previous disease (2); missing medical, radiological, and laboratory information (3); previous history of gastrointestinal diseases and bleeding; and (4) evidence of active gastrointestinal bleeding on admission.

### 2.2. Data collection

The baseline data collected for each patient included the following details (1): Demographic details such as age and sex (2); Medical history information such as current smoking, current drinking, hypertension, hyperlipidemia, diabetes mellitus, and heart disease (3); Location and maximum diameter of the aneurysm (4); Acute hydrocephalus (5); Clinical scales, including World Federation of Neurological Societies (WFNS) grade; modified Fisher scale (mFS) score, Graeb score, and Subarachnoid Hemorrhage Early Brain Edema score (SEBES) (6); Laboratory examination results, such as the monocyte count [normal range: (0.1–0.6) × 10^9^/L], lymphocyte count [normal range: (0.8–3.5) × 10^9^/L], neutrophil count [normal range: (2.0–7.5) × 10^9^/L], and white blood cell count [normal range [4-10]:×10^9^/L] (7); The mean HU value of the area supplied by the MCA (8); Treatment methods, including surgical clipping and endovascular coiling; and (9) In-hospital complications, including SRGB, delayed cerebral ischemia, anemia, pneumonia, and intracranial infection.

### 2.3. Image analysis

**Figure 1:**
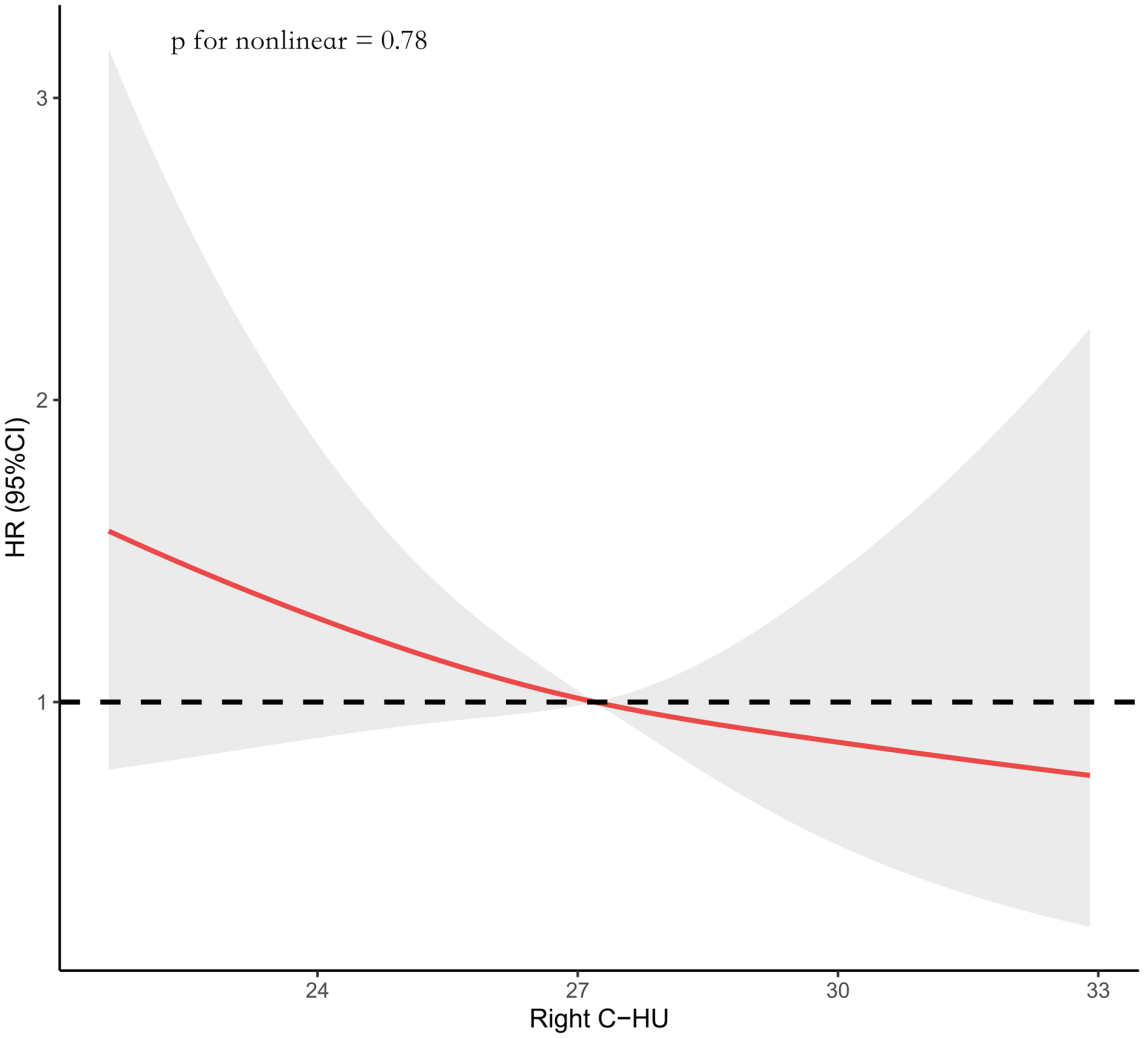
Data were fitted using a Cox regression model of the restricted cubic spline with three knots (the 10th, 50th, the 90th percentiles) for the right caudate Hounsfield Unit (Right C-HU) value. The model was adjusted for age, diabetes mellitus, posterior circulation, a SEBES score of 3–4, endovascular coiling, and left caudate HU values. The median right caudate HU value served as the reference.

Upon emergency admission, each patient underwent a standard non-contrast whole-head CT scan (with a 5-mm slice thickness) within the first 24 h. The unique Rapid system was utilized to delineate 10 distinct regions, which correspond to the standard MCA territory ASPECT areas, and to calculate the radiological density of the 10 separate regions using the HU value. The mean HU values for every ASPECT region were recorded (Figure 1).

The Rapid system is the first and only neuroimaging device that has been shown to improve reader diagnosis under the FDA’s CADx classification. It is based on the ASPECT score. It uses validated machine-learning algorithms to automatically identify regions of the brain and generate scores to help physicians quickly assess patient status.

### 2.4. Study outcomes

The primary outcome assessed in this study was the SRGB following aSAH. SRGB was specifically identified as the presence of fresh blood or ground coffee in nasogastric aspirate, hematemesis, melena, blood in the stool, positive occult blood test, or positive fecal occult blood test within 14 days of acute aSAH onset (4). For details of SRGB, please see Supplementary Table S2.

### 2.5. Statistical analysis

Continuous variables were presented as the mean ± standard deviation (SD) or median with interquartile range (IQR), and categorical variables as percentages. A Cox regression model was used for univariate and multivariate analyzes. All risk factors that showed a statistical trend (*p* < 0.05) in univariate analyzes were included in the multivariate Cox regression analysis with stepwise forward progression to identify risk factors independently associated with SRGB. The proportional hazard assumptions were assessed by examining Schoenfeld’s global test. Associations were expressed as the Hazard Ratio (HR) and 95% confidence interval (CI).

We evaluated the associations between the right caudate HU value at admission and the risk of developing SRGB by constructing a Cox regression model with a restricted cubic spline (RCS) (with three knots) for the right caudate HU value (continuous variable), adjusting for all potential covariates that showed a statistical trend in univariate analyzes.

The optimal cut-off value for the studied variables was determined with the R package “maxstat” (Maximally Selected Rank Statistics). We divided the patients into two groups according to the optimal cutoff point of the right caudate HU value. The Kaplan–Meier curves along with the log-rank test were used to visualize the cumulative risk of SRGB in the whole cohort with different risk factors. In sensitivity analyzes, we excluded patients with MCA aneurysm. Then we used the Cox regression model for univariate and multivariate analyzes.

To evaluate whether there was any significant interaction between these factors and the right caudate HU value when analyzing SRGB, we conducted subgroup analyzes stratified by age, sex, current smoking, current drinking, hypertension, WFNS grade, and treatment modality.

All statistical analyzes were performed with SPSS Statistics 26.0 (IBM, Armonk, New York, United States), R software (Version 4.2.1), and GraphPad PRISM 9.4.0 (GraphPad Software Inc., San Diego, CA, United States). Statistical significance was set at *p* < 0.05.

## 3. Results

### 3.1. Baseline characteristics

A total of 531 patients were included in the study. The clinical characteristics of the patients are summarized in Table 1. There were 95 (17.9%) patients with SRGB (Table 1).

**Table 1:** Clinical characteristics of the patients.

Variable	Cohort (*n* = 531)
Demographics	
Female, *n* (%)	324 (61.0)
Age > 65, *n* (%)	107 (20.2)
Medical history	
Current smoking, *n* (%)	57 (10.7)
Current drinking, *n* (%)	39 (7.3)
Hypertension, *n* (%)	270 (50.8)
Hyperlipidemia, *n* (%)	12 (2.3)
Diabetes mellitus, *n* (%)	30 (5.6)
Heart disease, *n* (%)	38 (7.2)
Aneurysm information	
Posterior circulation, *n* (%)	58 (10.9)
Maximum diameter of aneurysm^b^, mean ± SD	6.34 ± 3.87
Acute hydrocephalus, *n* (%)	208 (39.2)
Clinical scales	
WFNS grade 4–5, *n* (%)	113 (21.3)
mFS grade 3–4, *n* (%)	213 (40.1)
Graeb score 5–12, *n* (%)	34 (6.4)
SEBES score 3–4, *n* (%)	228 (42.9)
Admission laboratory results	
Monocyte count^a^, median (IQR)	0.39 (0.28–0.55)
Lymphocyte count^a^, median (IQR)	0.93 (0.69–1.26)
Neutrophil count^a^, median (IQR)	11.10 (8.57–13.78)
Leukocyte count^a^, median (IQR)	12.47 (9.95–15.07)
Mean HU value	
Right C, median (IQR)	27.20 (25.50–28.60)
Right IC, median (IQR)	26.40 (25.40–27.50)
Right L, median (IQR)	29.50 (28.30–30.50)
Right I, median (IQR)	28.50 (27.40–29.80)
Left C, median (IQR)	27.00 (25.30–28.30)
Left IC, median (IQR)	26.60 (25.60–27.50)
Left L, median (IQR)	29.50 (28.50–30.50)
Left I, median (IQR)	28.10 (27.00–29.40)
Treatment modality	
Surgical clipping, *n* (%)	308 (58.0)
Endovascular coiling, *n* (%)	223 (42.0)
In-hospital complications	
Stress-related gastrointestinal bleeding	95 (17.9)
Delayed cerebral ischemia	149 (28.1)
Anemia	224 (42.2)
Pneumonia	208 (39.2)
Intracranial infection	81 (15.3)

WFNS, World Federation of Neurological Societies; mFS, modified Fisher; SEBES, Subarachnoid Hemorrhage Early Brain Edema; C, caudate; IC, internal capsule; L, lentiform nucleus; I, insula; HU, Hounsfiled unit. ^a^Unit of measurement: 10^9^/L. ^b^Unit of measurement: mm.

### 3.2. The relationship between the caudate HU value and SRGB

As shown in Table 2, univariate analysis revealed that age, diabetes mellitus, posterior circulation, SEBES scores 3–4, right caudate HU values, left caudate HU values, and endovascular coiling were risk factors for SRGB in aSAH patients. The forward stepwise multivariate Cox regression analysis showed that endovascular coiling (HR = 4.448; 95% CI: 2.838–6.969; *p* < 0.001), and right caudate HU values (HR = 0.913; 95% CI: 0.847–0.983; *p* = 0.016) were independent risk factors associated with SRGB (Table 2).

**Table 2:** Univariate and multivariate analysis of SRGB.

Variable	Univariate				Multivariate	
	SRGB	No SRGB	HR (95% CI)	*p* value	HR (95% CI)	*P* value
No. of patients	95	436				
Demographics						
Female, *n* (%)	58 (61.1)	266 (61.0)	1.044 (0.691–1.577)	0.838		
Age > 65, *n* (%)	31 (32.6)	76 (17.4)	2.110 (1.374–3.241)	0.001		
Medical history						
Current smoking, *n* (%)	10 (10.5)	47 (10.8)	0.967 (0.502–1.862)	0.920		
Current drinking, *n* (%)	8 (8.4)	31 (7.1)	1.144 (0.555–2.360)	0.716		
Hypertension, *n* (%)	48 (50.5)	222 (50.9)	0.968 (0.647–1.447)	0.874		
Hyperlipidemia, *n* (%)	1 (1.1)	11 (2.5)	0.411 (0.057–2.950)	0.377		
Diabetes mellitus, *n* (%)	11 (11.6)	19 (4.4)	2.436 (1.299–4.570)	0.006		
Heart disease, *n* (%)	10 (10.5)	28 (6.4)	1.659 (0.861–3.196)	0.130		
Aneurysm information						
Posterior circulation, *n* (%)	23 (24.2)	35 (8.0)	3.166 (1.978–5.069)	<0.001		
Maximum diameter of aneurysm^b^, mean ± SD	6.49 ± 4.19	6.31 ± 3.80	1.005 (0.956–1.056)	0.852		
Acute hydrocephalus, n (%)	41 (43.2)	167 (38.3)	1.144 (0.762–1.718)	0.516		
Clinical scales						
WFNS grade 4–5, *n* (%)	28 (29.5)	85 (19.5)	1.493 (0.959–2.323)	0.076		
mFS grade 3–4, *n* (%)	64 (67.4)	254 (58.3)	1.366 (0.889–2.098)	0.154		
Graeb score 5–12, *n* (%)	9 (9.5)	25 (5.7)	1.578 (0.794–3.136)	0.193		
SEBES score 3–4, *n* (%)	32 (33.7)	196 (45.0)	0.617 (0.403–0.944)	0.026		
Admission laboratory results						
Monocyte count^a^, median (IQR)	0.4 (0.29–0.54)	0.39 (0.28–0.55)	1.266 (0.490–3.269)	0.626		
Lymphocyte count^a^, median (IQR)	0.94 (0.67–1.22)	0.93 (0.69–1.28)	1.037 (0.722–1.490)	0.844		
Neutrophil count^a^, median (IQR)	10.86 (8.11–13.84)	11.14 (8.65–13.72)	0.973 (0.922–1.026)	0.310		
Leukocyte count^a^, median (IQR)	12.20 (9.42–15.26)	12.55 (10.06–15.07)	0.974 (0.924–1.028)	0.338		
Mean HU value						
Right C, median (IQR)	26.60 (24.60–27.90)	27.30 (25.80–28.60)	0.888 (0.823–0.958)	0.002	0.913 (0.847–0.983)	0.016
Right IC, median (IQR)	26.50 (25.00–27.50)	26.40 (25.50–27.40)	0.938 (0.835–1.053)	0.279		
Right L, median (IQR)	29.50 (28.00–30.60)	29.50 (28.30–30.50)	0.950 (0.865–1.043)	0.279		
Right I, median (IQR)	28.40 (27.00–29.80)	28.60 (27.40–29.90)	0.938 (0.863–1.019)	0.130		
Left C, median (IQR)	26.70 (24.20–27.80)	27.00 (25.60–28.40)	0.923 (0.862–0.988)	0.021		
Left IC, median (IQR)	26.80 (25.20–27.70)	26.60 (25.70–27.50)	0.936 (0.837–1.045)	0.239		
Left L, median (IQR)	29.50 (27.90–30.60)	29.55 (28.60–30.48)	0.921 (0.831–1.020)	0.114		
Left I, median (IQR)	28.10 (26.60–29.60)	28.20 (27.10–29.40)	0.970 (0.899–1.047)	0.434		
Treatment modality						
Endovascular coiling, *n* (%)	67 (70.5)	156 (35.8)	4.656 (2.977–7.282)	<0.001	4.448 (2.838–6.969)	<0.001

SRGB, stress-related gastrointestinal bleeding; WFNS, World Federation of Neurological Societies; mFS, modified Fisher; SEBES, Subarachnoid Hemorrhage Early Brain Edema; C, caudate; IC, internal capsule; L, lentiform nucleus; I, insula; HU, Hounsfiled unit. ^a^Unit of measurement: 10^9^/L. ^b^Unit of measurement: mm.

**Figure 2:**
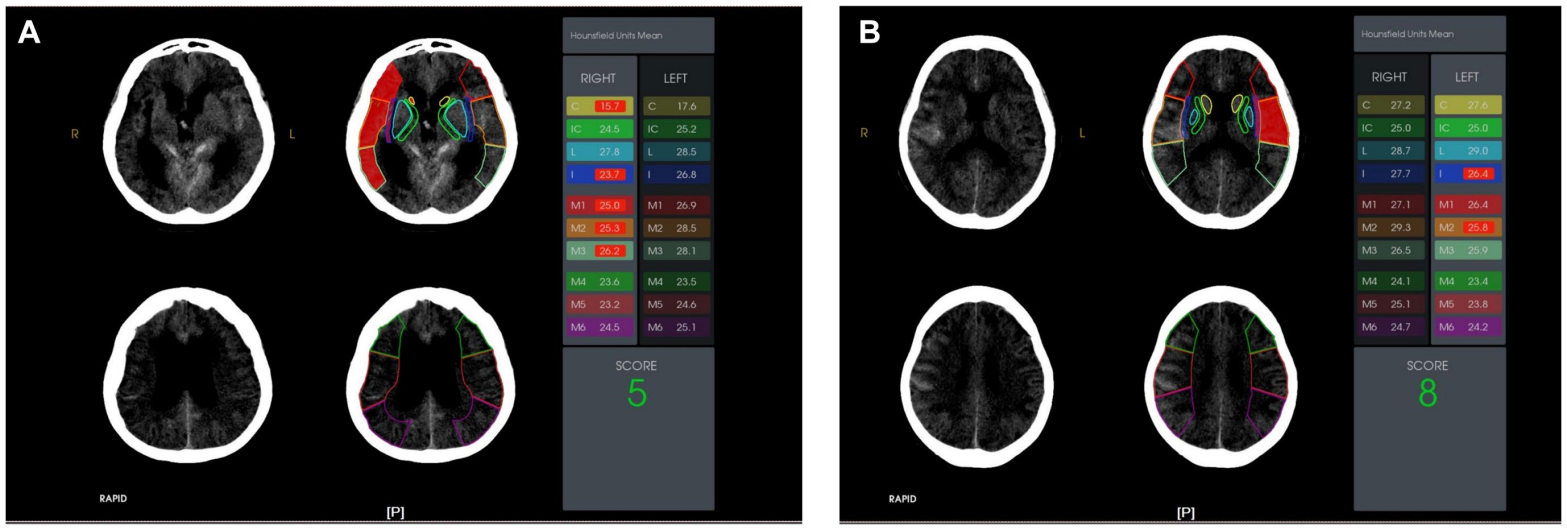
The ten Alberta Stroke Program Early Computer Tomography (ASPECT) score regions were outlined on the non-contrast computed tomography head images. The Hounsfield Unit (HU) value difference in the ten ASPECT score regions are shown. Case presentations: **(A)** A 62-year-old female with a right caudate HU value of 15.7 on admission. She suffered from stress-related gastrointestinal bleeding after an aneurysmal subarachnoid hemorrhage; **(B)** A 43-year-old male with a right caudate HU value of 27.2 on admission. He did not suffer from stress-related gastrointestinal bleeding after aneurysmal subarachnoid hemorrhage.

Using the Cox regression model with RCS, we found that the incidence of developing SRGB reduces with increasing right caudate HU values (p for nonlinear = 0.78; Figure 2).

**Figure 3:**
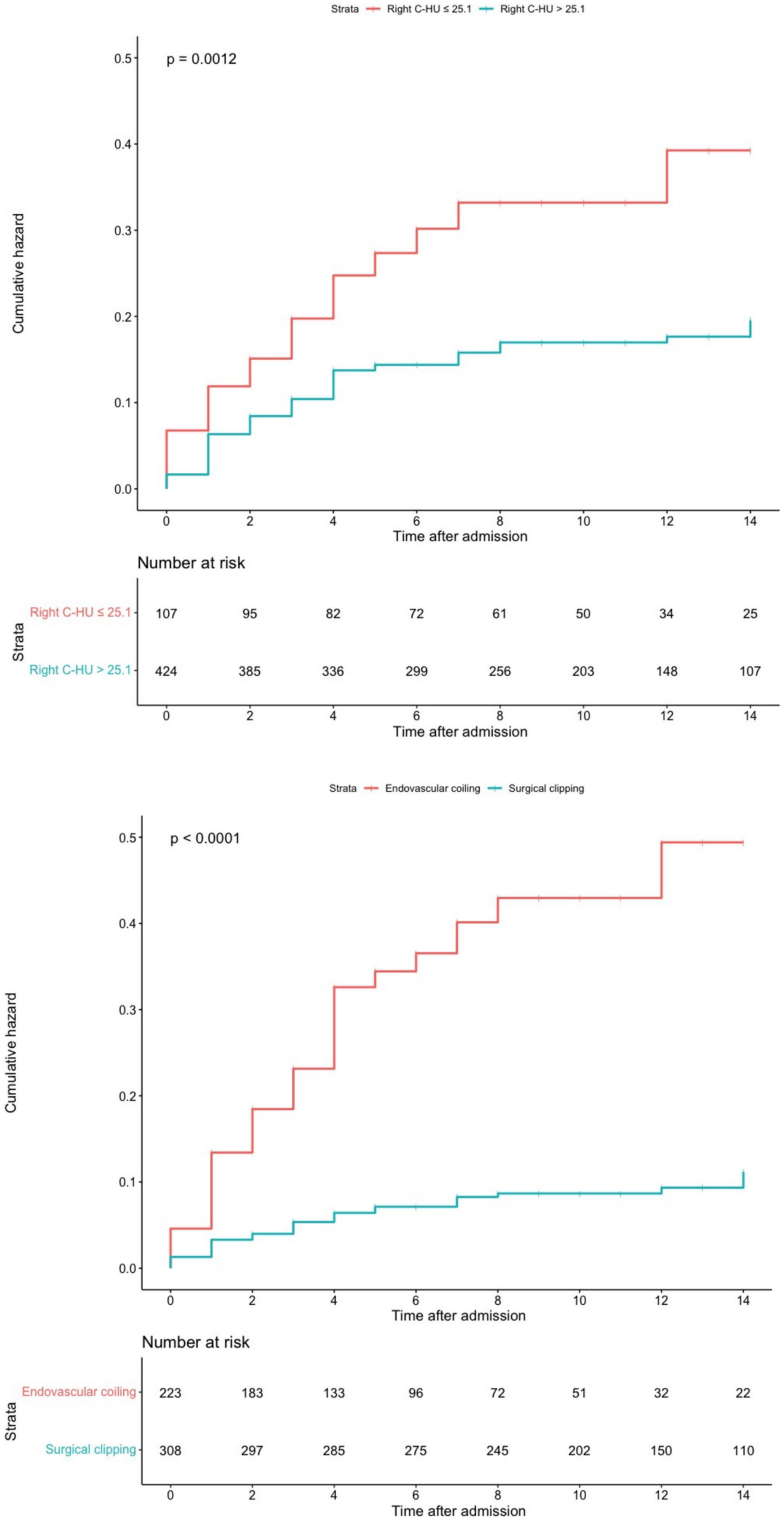
Kaplan–Meier curves and log-rank test for stress-related gastrointestinal bleeding.

The optimal cut-off point for the right caudate HU value was 25.1. The cumulative SRGB rate was higher in the group with right caudate HU values ≤25.1 than in the group with right caudate HU values > 25.1 (log-rank, *p* = 0.0012). In addition, compared with the surgical clipping group, the cumulative SRGB rate was higher in the endovascular coiling group (log-rank, *p* < 0.0001; Figure 3).

The sensitivity analysis which excluded participants with MCA aneurysm did not substantially alter the association between the right caudate HU value and SRGB (Supplementary Table S1).

### 3.3. Subgroup analysis

Subgroup analysis showed no significant interactions among subgroups for age, sex, current smoking, current drinking, hypertension, WFNS grade, and treatment modality (Table 3).

**Table 3:** Subgroup analysis of associations between right caudate HU values and SRGB.

Subgroup	Right C HU value	No. of patients	Events, *n* (%)	Hazard Ratio (95% CI)	*p* for interaction
Age					0.104
≤65	Q1	356	44 (12.4)	1 [Reference]	
	Q2	68	20 (29.4)	1.673 (0.839–3.338)	
>65	Q1	68	20 (29.4)	1 [Reference]	
	Q2	39	11 (28.2)	0.873 (0.371–2.056)	
Sex					0.714
Male	Q1	168	25 (14.9)	1 [Reference]	
	Q2	39	12 (30.8)	1.119 (0.460–2.722)	
Female	Q1	256	39 (15.2)	1 [Reference]	
	Q2	68	19 (27.9)	1.676 (0.846–3.319)	
Current smoking					0.491
Yes	Q1	49	7 (14.3)	1 [Reference]	
	Q2	8	3 (37.5)	1.873 (0.350–10.022)	
No	Q1	375	57 (15.2)	1 [Reference]	
	Q2	99	28 (28.3)	1.378 (0.775–2.451)	
Current drinking					0.716
Yes	Q1	32	7 (21.9)	1 [Reference]	
	Q2	7	1 (14.3)	0.170 (0.012–2.424)	
No	Q1	392	57 (14.5)	1 [Reference]	
	Q2	100	30 (30.0)	1.538 (0.883–2.678)	
Hypertension					0.060
Yes	Q1	213	34 (16.0)	1 [Reference]	
	Q2	57	14 (24.6)	0.858 (0.371–1.985)	
No	Q1	211	30 (14.2)	1 [Reference]	
	Q2	50	17 (34.0)	2.503 (1.202–5.213)	
WFNS grade					0.248
1–3	Q1	343	47 (13.7)	1 [Reference]	
	Q2	75	20 (26.7)	1.408 (0.692–2.862)	
4–5	Q1	81	17 (21.0)	1 [Reference]	
	Q2	32	11 (34.4)	0.999 (0.380–2.627)	
Treatment modality					0.530
SC	Q1	255	22 (8.6)	1 [Reference]	
	Q2	53	6 (11.3)	0.714 (0.232–2.195)	
EC	Q1	169	42 (24.9)	1 [Reference]	
	Q2	54	25 (46.3)	1.669 (0.872–3.196)	

Q1, right caudate HU value ≥ 25.1; Q2, right caudate HU value < 25.1, WFNS, World Federation of Neurological Societies; SC, surgical clipping; EC, endovascular coiling.

## 4. Discussion

In the current study, we have identified the predictive effect of the right caudate HU value in forecasting the development of SRGB following aSAH. Our main findings were as follows (1): the overall incidence of SRGB was 17.9% in our single-center cohort (2); endovascular coiling and right caudate HU values were predictors for SRGB (3); a HU value ≤25.1 in the right caudate was the predictive cutoff value for SRGB.

The HU value in the ASPECT score system quantitatively reflects the degree of tissue X-ray absorption, reliably detecting ischemic changes in brain tissue (14). Previous research on the ASPECT score has primarily focused on ischemic stroke in the MCA territory. Although aSAH is a hemorrhagic stroke, the main risk factor of this disease is ischemia. It has been found that the ASPECT score has important clinical application value for aSAH. Specifically, it significantly correlates with functional outcomes in patients with delayed cerebral ischemia following aSAH (1). Notably, our sensitivity analysis excluded patients with MCA aneurysm and still observed that the right caudate HU value significantly correlated with the development of SRGB, emphasizing the broader utility of the ASPECT score as a clinical tool.

SRGB refers to acute lesions of the gastrointestinal system in patients suffering from serious injuries, illnesses, and other stressful situations. In the process of SRGB, the metabolism of central monoamine neurotransmitters is accelerated. Norepinephrine, dopamine, γ-aminobutyric acid, serotonin, and acetylcholine may all be involved in the induction of SRGB. Previous research indicated that dopamine plays a significant role in gastric mucosal erosions. The central nervous system has three major dopaminergic pathways: the nigrostriatal pathway, mesolimbic pathway, and mesocortical pathway (15). First, the nigrostriatal pathway originates from the SN and projects to the caudate putamen and the central nucleus of the amygdala (10, 16). Second, the mesolimbic pathway receives input from neurons in the lateral ventral tegmental area (VTA) and projects to the nucleus accumbens, olfactory tubercle, septum, amygdala, and hippocampus (9). Eventually, the mesocortical pathway also descends from the VTA but includes ascending projections that follow a more medial route. Research has revealed that bilateral microinfusions of N-methyl-D-aspartate (NMDA), a highly specific neurotoxic agent, into the substantia nigra (SN) led to gastric erosions 24 h after surgery. Furthermore, a decrease in dopamine levels within the caudate 24 h after the NMDA infusion into the SN was observed (15). Additionally, Strang found that the incidence of duodenal ulcers is higher in Parkinson’s disease patients than in the general population. This phenomenon is primarily attributed to decreased dopamine and/or hypofunction in the substantial nigra-striatum of the central nervous system (17). Therefore, we hypothesize that hypoperfusion of the caudate nucleus following aSAH contributes to the hypofunctioning dopaminergic system, which plays a crucial role in the pathogenesis of SRGB. Our study demonstrated no effect of the left caudate HU value on SRGB in the multivariate Cox regression analysis. However, this phenomenon may be due to the different functions of the left and right insula. The issue requires further study.

In a previous study involving 627 patients with aSAH, the overall incidence of gastrointestinal bleeding was reported to be 4.9% (18). However, in our study, the incidence rate of SRGB was notably higher, at 17.9%. This trend aligns with findings regarding the incidence of SRGB after intracranial hemorrhage, which has widely varying incidence rates, ranging from low (0.3%) to high (32.3%). The discrepancies may be attributed to the varied ways in which SRGB is defined and documented across studies. For instance, in a previous study, the authors defined SRGB as the occurrence of any of the following events within 24 h of reported gastrointestinal bleeding (1): blood transfusion requirement (2); hemoglobin reduction of 2 gm/dl or greater; or (3) an abrupt systolic blood pressure reduction of ≥20 mmHg (18). Furthermore, regional disparities, differences in medical resources and care, and varying physician perspectives may further contribute to these differences.

aSAH often necessitates complex, multimodal treatment delivered by a diverse range of medical professionals. Currently, endovascular intervention (EVI) has emerged as the primary treatment modality. However, EVI carries a risk of thromboembolic events, which are estimated to occur in 2.4–5.2% of cases. Consequently, antiplatelet medications are frequently administered during endovascular treatment to prevent thromboembolism (19). Unfortunately, this preventative mechanism comes at the cost of a heightened risk of SRGB. Prostaglandins are essential for protecting the gastric mucosa. They increase mucosal blood flow, promote the proliferation of gastric epithelial cells, and stimulate mucus and bicarbonate secretion. Therefore, inhibition of prostaglandin synthesis by antiplatelet drugs makes gastric mucosa prone to ulceration and bleeding in a highly acidic environment. Furthermore, the acidic environment in the stomach causes aspirin to remain non-ionized, allowing it to accumulate in the gastric mucosal cells. It can alter the cells’ permeability and lead to ulceration (20). The use of anticoagulant or antiplatelet drugs has been listed as a risk factor for SRGB. Thus, it seems reasonable to assume that patients treated with endovascular coiling have an increased risk of SRGB.

Our study is the first study to demonstrate the clinical value of using the right caudate HU value in the ASPECT score to predict SRGB in patients with aSAH. It is easily obtained by the Rapid system in clinical practice. Therefore, our research findings can be used for patient care and preoperative assessment by neurosurgeons, intensive care unit (ICU) physicians, and other medical personnel. With early detection, doctors can take early preventive medication. In addition, the correlation between the right caudate HU value and SRGB provides further clinical evidence of the interplay between dopaminergic pathways and the gastrointestinal tract.

### 4.1. Limitations

Our study has some limitations (1): Previous study has shown that the ASPECT score has different weights for each region (21). We did not weigh each region when calculating the HU difference. (2) The population we studied came from northern China, and it is unclear whether racial differences exist in this phenomenon. (3) This study was conducted in only one center, which may make it subject to selection bias. Further prospective multicenter studies are needed to validate our results.

## 5. Conclusion

In this large observational cohort, the right caudate HU value is a predictor for SRGB of aSAH patients. Our findings provide further evidence for the relationship between the gastrointestinal system and the brain.

## Data availability statement

The original contributions presented in the study are included in the article/Supplementary material, further inquiries can be directed to the corresponding authors.

## Ethics statement

This study received ethical approval from the Institutional Review Board of Beijing Tiantan Hospital (KY 2021–008-01). The studies were conducted in accordance with the local legislation and institutional requirements. Written informed consent for participation was required from the participants or the participants’ legal guardians/next of kin in accordance with the national legislation and institutional requirements.

## Author contributions

XC and YuanZ: conception, design, and reviewed submitted version of manuscript. KY, KW, RunL, FL, YC, HH, and RuiL: acquisition of data. KW and KY: drafting the article. KY, FL, YC, and QH: statistical analysis. YJ, YunZ, and TL: administrative, technical, and material support. XC, and YuanZ: study supervision. All authors, analysis, interpretation of data, contributed to the article, and approved the submitted version.

## Funding

This study was supported by the National Key Research and Development Program of China (Grant Nos. 2021YFC2501101 and 2020YFC2004701), the National Natural Science Foundation of China (Grant Nos. 82071296, 81671129, and 81471210), the Beijing Municipal Administration of Hospitals Incubating Program, Beijing, China (Grant No. pX2020023), and the Natural Science Foundation of Beijing, China (Grant No. 7204253).

## Conflict of interest

The authors declare that the research was conducted in the absence of any commercial or financial relationships that could be construed as a potential conflict of interest.

## Publisher’s note

All claims expressed in this article are solely those of the authors and do not necessarily represent those of their affiliated organizations, or those of the publisher, the editors and the reviewers. Any product that may be evaluated in this article, or claim that may be made by its manufacturer, is not guaranteed or endorsed by the publisher.
