# Supplementary material for: Prediction of stress-related gastrointestinal bleeding in patients with aneurysmal subarachnoid hemorrhage using caudate Hounsfield unit value in ASPECT score
Source: Front Neurol. 2023 Sep 13;14:1237310. doi: 10.3389/fneur.2023.1237310 (PMC10533991; doi:10.3389/fneur.2023.1237310)
Supplement: Supplementary file 1 [file Table_1.pdf]

Table S1. Univariate and Multivariate Analysis of SRGB in Patients with Non-middle Cerebral Aneurysm

Variable	Univariate				Multivariate	
	SRGB	No SRGB	HR (95% CI)	P value	HR (95% CI)	P value
No. of patients	82	339				
Demographics						
Female, n (%)	29 (35.4)	214 (63.1)	1.090 (0.693-1.714)	0.709		
Age>65, n (%)	26 (31.7)	59 (17.4)	2.019 (1.268-3.216)	0.003		
Medical history						
Current smoking, n (%)	8 (9.8)	37 (10.9)	0.889 (0.428-1.843)	0.751		
Current drinking, n (%)	7 (8.5)	23 (6.8)	1.207 (0.556-2.619)	0.634		
Hypertension, n (%)	41 (50.0)	172 (50.7)	0.961 (0.623-1.482)	0.858		
Hyperlipidemia, n (%)	1 (1.2)	7 (2.1)	0.581 (0.081-4.177)	0.590		
Diabetes mellitus, n (%)	10 (12.2)	16 (4.7)	2.312 (1.192-4.483)	0.013		
Heart disease, n (%)	9 (11.0)	25 (7.4)	1.514 (0.757-3.027)	0.241		
Aneurysm information						
Posterior circulation, n (%)	23 (28.0)	35 (10.3)	2.870 (1.771-4.652)	<0.001		
Maximum diameter of aneurysm[b], mean ± SD	6.05±3.80	5.89±3.41	1.008 (0.949-1.070)	0.806		
Clinical scales						
WFNS grade 4-5, n (%)	23 (28.0)	52 (15.3)	1.794 (1.107-2.907)	0.018	0.580 (0.355-0.948)	0.030
mFS grade 3-4, n (%)	27 (32.9)	148 (43.7)	1.467 (0.926-2.326)	0.103		
Graeb score 5-12, n (%)	8 (9.8)	24 (7.1)	1.322 (0.637-2.744)	0.453		
SEBES score 3-4, n (%)	26 (31.7)	146 (43.1)	0.616 (0.387-0.981)	0.041		
Admission laboratory results						
Monocyte count[a], median (IQR)	0.40 (0.29-0.53)	0.38 (0.27-0.53)	1.679 (0.596-4.729)	0.327		

Lymphocyte count[a], median (IQR)	0.93 (0.69-1.19)	0.92 (0.69-1.26)	1.043 (0.696-1.565)	0.837		
Neutrophil count[a], median (IQR)	10.90 (7.91-13.80)	11.23 (8.72-13.78)	0.976 (0.920-1.035)	0.409		
Leukocyte count[a], median (IQR)	12.28 (9.42-15.06)	12.65 (10.15-15.05)	0.978 (0.922-1.037)	0.457		
Mean HU value						
Right C, median (IQR)	26.35 (24.30-27.73)	27.20 (25.70-28.60)	0.883 (0.816-0.957)	0.002	0.919 (0.849-0.995)	0.038
Right IC, median (IQR)	26.50 (24.78-27.50)	26.30 (25.50-27.30)	0.925 (0.811-1.055)	0.244		
Right L, median (IQR)	29.50 (27.80-30.60)	29.40 (28.30-30.40)	0.933 (0.833-1.045)	0.230		
Right I, median (IQR)	28.20 (26.88-29.63)	28.40 (27.30-29.60)	0.936 (0.845-1.036)	0.203		
Left C, median (IQR)	26.10 (24.10-27.53)	26.90 (25.50-28.30)	0.905 (0.843-0.971)	0.005		
Left IC, median (IQR)	26.55 (24.98-27.53)	26.50 (25.70-27.40)	0.913 (0.805-1.036)	0.157		
Left L, median (IQR)	29.40 (27.68-30.43)	29.40 (28.60-30.40)	0.905 (0.805-1.017)	0.093		
Left I, median (IQR)	27.90 (26.60-29.40)	28.10 (27.00-29.20)	0.965 (0.869-1.072)	0.510		
Treatment modality						
Endovascular coiling, n (%)	63 (76.8)	147 (43.4)	4.569 (2.718-7.680)	<0.001	4.377 (2.599-7.373)	<0.001

Abbreviations: SRGB: stress-related gastrointestinal bleeding; WFNS: World Federation of Neurological Societies; mFS: modified Fisher; SEBES: Subarachnoid Hemorrhage Early Brain Edema; C: caudate; IC: internal capsule; L: lentiform nucleus; I: insula; HU: Hounsfiled unit

[a]Unit of measurement: 10^9/L

[b]unit of measurement: mm

Table S2. Symptoms of SRGB

Category of symptoms	Cohort (n=95)
Positive occult blood test	50 (52.6)
Melena	21 (22.1)
Blood in the stool	11 (11.6)
Presence of fresh blood or ground coffee in nasogastric aspirate	10 (10.5)
Hematemesis	3 (3.2)
